# Southern Medical College Hospital

**Published:** 1882-06-20

**Authors:** 


### *SOUTHERN MEDICAL COLLEGE HOSPITAL.*

The Trustees of the Southern Medical College, Atlanta, have secured the Central Hotel property, on Ivy street, for hospital purposes, to be under the medical supervision of the faculty of said institution. It is being now rapidly repaired and fitted up, and will go into speedy operation. The building is a large one, and the lot on which it stands connects with the college lot, and will be very convenient to the students of the Southern Medical College, to whom it will give special advantages in the acquirement of practical instruction in the line of their studies.

